# Implementation of Best-Evidence Osteoarthritis Care: Perspectives on Challenges for, and Opportunities From, Low and Middle-Income Countries

**DOI:** 10.3389/fresc.2021.826765

**Published:** 2022-01-24

**Authors:** Jillian P. Eyles, Saurab Sharma, Rosa Weiss Telles, Mosedi Namane, David J. Hunter, Jocelyn L. Bowden

**Affiliations:** ^1^Kolling Institute of Medical Research, Faculty of Medicine and Health, University of Sydney, Sydney, NSW, Australia; ^2^Centre for Pain IMPACT, Neuroscience Research Australia, Sydney, NSW, Australia; ^3^Universidade Federal de Minas Gerais, Brazilian Longitudinal Study of Adult Health (ELSA-Brasil) Musculoskeletal, Belo Horizonte, Brazil; ^4^School of Public Health and Family Medicine, The University of Cape Town, Cape Town, South Africa; ^5^Rheumatology Department, Royal North Shore Hospital, Sydney, NSW, Australia

**Keywords:** osteoarthritis, health inequities, recommended care, low- and middle-income countries (LMICs), implementation

## Abstract

The “Joint Effort Initiative” (JEI) is an international consortium of clinicians, researchers, and consumers under the auspices of the Osteoarthritis Research Society International (OARSI). The JEI was formed with a vision to improve the implementation of coordinated programs of best evidence osteoarthritis care globally. To better understand some of the issues around osteoarthritis care in low- and middle-income countries (LMICs), the JEI invited clinician researcher representatives from South Africa, Brazil, and Nepal to discuss their perspectives on challenges and opportunities to implementing best-evidence osteoarthritis care at the OARSI World Pre-Congress Workshop. We summarize and discuss the main themes of the presentations in this paper. The challenges to implementing evidence-based osteoarthritis care identified in LMICs include health inequities, unaffordability of osteoarthritis management and the failure to recognize osteoarthritis as an important disease. Fragmented healthcare services and a lack of health professional knowledge and skills are also important factors affecting osteoarthritis care in LMICs. We discuss considerations for developing strategies to improve osteoarthritis care in LMICs. Existing opportunities may be leveraged to facilitate the implementation of best-evidence osteoarthritis care. We also discuss strategies to support the implementation, such as the provision of high-quality healthcare professional and consumer education, and systemic healthcare reforms.

## Introduction

The World Health Organization acknowledges osteoarthritis, a chronic disease that affects the tissues of moveable joints, as a leading cause of disability and a major threat to healthy aging (1). Osteoarthritis has significant impacts on morbidity, mortality, quality-of-life, and increases the risks of poverty. Poverty is not only the lack of sustainable income for basic necessities (food, shelter, education, healthcare), it also refers to limited capacity to participate effectively in society, and importantly, it may lead to social discrimination and exclusion from participation in decision-making (2). Osteoarthritis is thought to impose a greater burden for those living in low- and middle-income countries (LMICs) by creating a vicious cycle of pain and disability that subsequently worsens these outcomes (3). The global prevalence of hip and knee osteoarthritis is estimated at 3754.2 per 100,000 population (4). The prevalence of osteoarthritis in LMICs fluctuates around the global estimate, and is rising. For example, the prevalence of osteoarthritis in South Africa, Brazil, and Nepal has risen by 9, 14, and 20%, respectively, between 2010 and 2017 (4). During the same period, estimates of years lived with disability (YLD) attributed to osteoarthritis increased by 10% globally, while estimates rose by 9% in South Africa, 15% in Brazil, and 21% in Nepal (4). Despite increasing prevalence rates and YLDs, little is known about the determinants of osteoarthritis health in LMICs (5, 6), or how to best tackle this urgent public health problem. These issues have been highlighted as a priority area for international osteoarthritis research (7).

The key components of first-line, best evidence care for osteoarthritis are education and support for self-management, physical activity, and exercise and maintaining healthy bodyweight (8). Many people with osteoarthritis in high-income countries are still not receiving these first-line treatments, and are missing out on the care they need to fully live their lives (9). This situation is amplified in LMICs as both health system-level and individual-level factors influence access to care (10–12). While many challenges to implementing osteoarthritis care are similar globally, it is recognized that LMICs face challenges and needs specific to their local contexts. Little is known about these challenges and needs, although several issues have been highlighted: inequity of care; costs of delivering and receiving treatment; and lack of training for health professionals (10, 12).

The “Joint Effort Initiative” (JEI), an international consortium of clinicians, researchers, and consumers under the auspices of the Osteoarthritis Research Society International (OARSI), was formed with a vision to improve the implementation of coordinated programs of best evidence osteoarthritis care globally (13). To better understand some of the issues around osteoarthritis care in LMICs, the JEI invited clinician researchers from South Africa, Brazil, and Nepal to discuss their perspectives on challenges and opportunities to implementing best-evidence osteoarthritis care at the OARSI World Pre-Congress Workshop “Implementing osteoarthritis management programs around the world” April 28th, 2021. This paper summarizes their presentations, supported by relevant evidence from the literature.

The Global Alliance for Musculoskeletal Health (GMUSC) is a network of international patient, professional, scientific, and civil society organizations focused on the prioritization musculoskeletal health. GMUSC has developed a blueprint to strengthen health systems for musculoskeletal health with eight strategic priority areas (pillars) which include (14):

1. Engagement, empowerment, and education of people, communities, governments.
2. Leadership, governance, and accountability.
3. Financial considerations.
4. Delivery of health services.
5. Equitable access to medicines and technologies.
6. Workforce.
7. Monitoring population health.
8. Research and health innovation.

We used the eight pillars as a framework to discuss future considerations for the implementation of best evidence osteoarthritis care in Brazil, South Africa, and Nepal.

## Methods

The presenters from Africa, South America, and Asia (MN, RWT, and SS) were chosen by the Steering Committee of the JEI as they were clinician-researchers, experienced in treating people with osteoarthritis, with interests in research. The cumulative clinical and research experience of the presenters was 46 years. The presenters were asked to address the following questions:

1. How are people with osteoarthritis in your country/region usually managed?
2. What challenges and opportunities are there for implementing programs that deliver best evidence osteoarthritis care in your country/region?
3. What contextual factors (e.g., system/political, cultural, and individual) should be considered when developing strategies to deliver best evidence osteoarthritis care in your country/region?

The presenters also took part in a panel discussion. The presentations and panel discussion were recorded, transcribed, and analyzed thematically by two researchers (JE and JB) who independently read the transcriptions and categorized the text into themes. The two researchers then met to discuss the transcription line-by-line and agreed on the main themes. The themes identified were explored through a review of the literature. Medline was searched using combinations of terms including “osteoarthritis,” “musculoskeletal,” “LMIC,” “developing countries,” “low- and middle-income countries,” “South Africa,” “Brazil,” “Nepal,” and “healthcare disparities.” Key articles of importance were also selected from the authors' prior knowledge of the literature and reference lists of key articles.

## Results

### Barriers to Best Evidence Osteoarthritis Care

**Figure 1:**
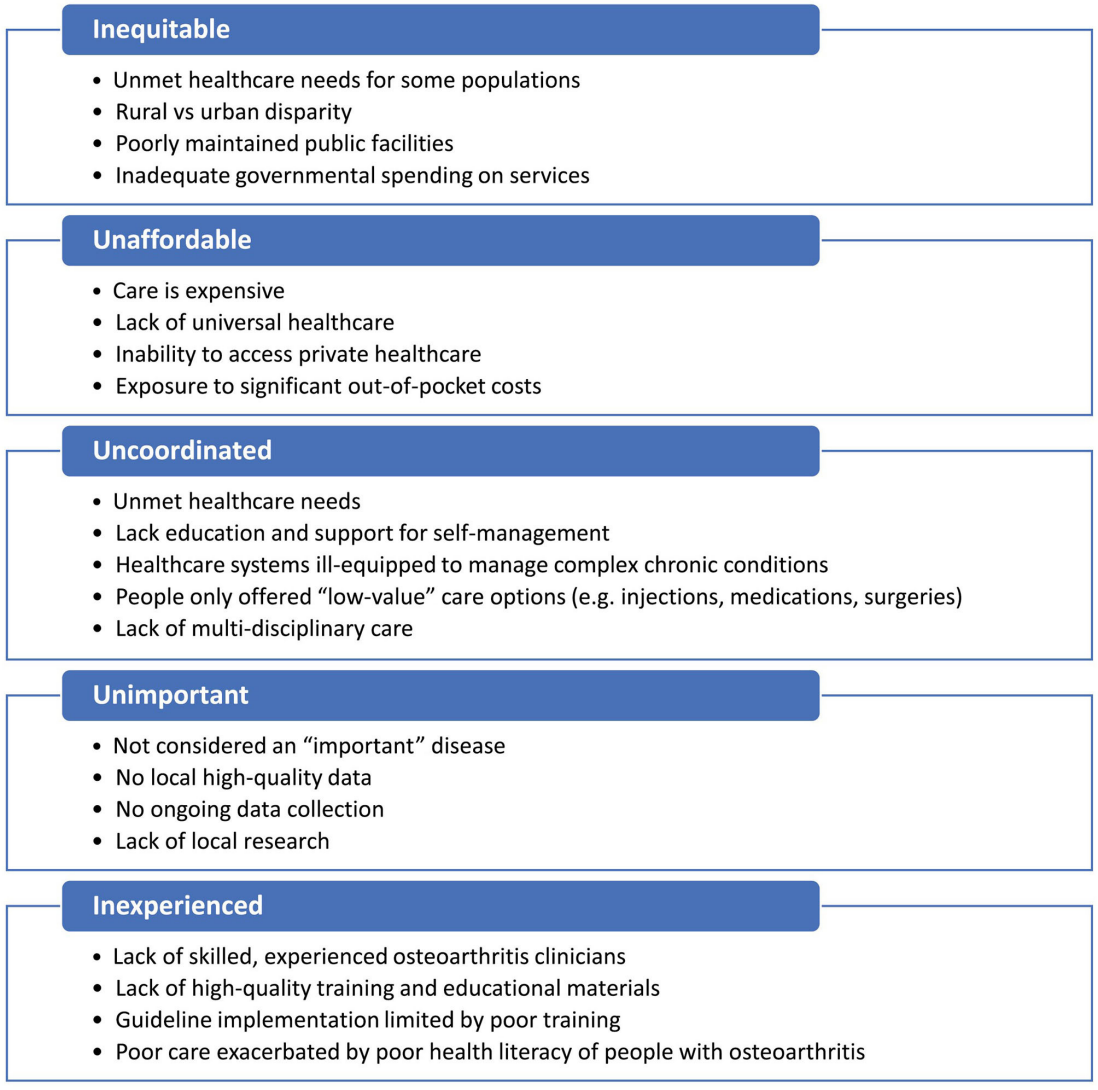
Barriers to high-value osteoarthritis care in Low- and Middle-Income Countries.

There were five main themes concerning barriers to best evidence osteoarthritis care (summarized in Figure 1):

*l*. *Health inequities*

Health inequity refers to differences in opportunity to attain full health potential by different groups of people, usually defined socially, economically, demographically, or geographically (15). There are strong relationships between health and wealth in South Africa, Brazil, and Nepal where health inequities are associated with high levels of poverty and exacerbated by inadequate levels of health insurance.

South Africa is currently working toward implementing a National Health Insurance program (16). Currently, only primary care in the public sector is provided free-of-charge and 84% of people depend on it for their healthcare (17). In Brazil the Sistema Único de Saúde (SUS) is a public health system that covers all levels of health, it provides healthcare for 75% of the population. In both countries, low levels of government spending on health have left the facilities and infrastructure run-down. This, coupled with inadequate spending on the delivery of health services, has contributed to substantial unmet needs (17, 18). In Nepal, the public health system offers universal access to basic emergency and in-patient services, and 40 essential drugs (19). Beyond this, out-of-pocket expenditure is the principal means of financing healthcare (19, 20). Consequently, there are high levels of unmet health needs for many in Nepal, especially for older people (21), and musculoskeletal health is particularly poorly serviced.

Health inequities in rural areas within LMICs are particularly stark. In South Africa, access to healthcare is more limited in rural areas where health personnel are often limited to students, new graduates, and medical aides (22). A qualitative study of people with knee osteoarthritis in rural Western Cape revealed three themes: lack of osteoarthritis education, barriers to osteoarthritis-related healthcare, and physical restrictions, such as reduction in mobility and inability to do household chores, that lowered quality-of-life (12). Further, South African provinces are governed by different political parties, which impacts the care available in each region, and results in fragmentation of health services (17). The access to health services across different regions in Brazil is also heterogeneous. It is estimated that >18% of people in Brazil have poor access to healthcare, which increases to >32% in rural areas (23). Access is also worse for people from minority ethnic groups, those without schooling and people from lower socioeconomic strata (23). In Nepal, many people with osteoarthritis in rural areas simply do not receive any treatment.

*ll*. *Unaffordability of osteoarthritis care*

Although much of the osteoarthritis care provided in South Africa is in the public primary health clinics, due to poor resourcing there are long waiting times and little support to assist people to self-manage their osteoarthritis (12). People in Brazil often pay for their own osteoarthritis treatment (24), as SUS services are limited and the waiting times are also long (25). To access osteoarthritis care in Nepal, one must have health insurance or the capacity to pay (19, 20). As healthcare in Nepal is very expensive, many people simply go without.

*lll*. *Lack of coordinated osteoarthritis care and overuse of low-value care options*

Healthcare systems of LMICs are generally ill-equipped to manage complex chronic conditions and support people in self-management (26). Where healthcare is available, the treatments offered often represent low-value care (27). In South Africa, the first point of contact for most patients are public sector primary care clinics which are nurse-driven and supported by physicians. There is a lack of specific coordinated osteoarthritis care which is more pronounced in rural areas.

In Brazil, people with osteoarthritis can be referred to medical specialists, but referrals to allied health professionals are less common. Osteoarthritis management is not coordinated or multidisciplinary and is often not evidence-based (28, 29).

Urban Nepali people with osteoarthritis are primarily managed by orthopedic surgeons and contrary to recommended care, are commonly offered low-value options such as injections, medications, surgeries, and advice to avoid activities. Osteoarthritis is increasingly managed by pain physicians who take a biomedical approach, providing expensive and unproven invasive therapies such as radiofrequency ablations, stem-cell therapies, and protein-rich plasma therapy (30, 31).

*lV*. *Not seen as an important condition and lack of high-quality local osteoarthritis data*

Osteoarthritis is a leading cause of disability (32) but has no direct impact on mortality rate. In LMICs, where health budgets are limited, osteoarthritis is neglected. There is a dearth of published osteoarthritis research and no ongoing national data collection for osteoarthritis in in South Africa, Brazil, and Nepal. Some positive changes have been seen recently, for example, data related to chronic pain was recently collected in South Africa via a national health survey (33); the Brazilian Study of Adult Health Musculoskeletal cohort (ELSA-Brasil MSK) (34) will provide important data about osteoarthritis and multimorbidity and an item on non-specific “joint pain” has been included in the World Health Organization STEPwise approach to surveillance survey in Nepal (35).

In LMICs, as in other parts of the world, other non-communicable diseases (NCDs) such as cardiovascular and respiratory diseases, trauma/injuries, and cancers are prioritized over musculoskeletal conditions such as osteoarthritis, despite the enormous burden associated with them (36). Osteoarthritis is one component of the “musculoskeletal pattern” of multimorbidity, commonly affecting people with comorbid cardiovascular and metabolic NCDs (37). It shares common risk factors and possible causal pathways with other NCDs, which are under-investigated around the world, especially in LMICs. This is an important area for future work, and may create exciting opportunitities for leveraging chronic disease management across common NCDs.

*V*. *Lack of skilled, experienced staff, and high-quality educational resources*

Despite the availability of evidence-based national clinical practice guidelines (CPGs) for osteoarthritis in South Africa (38), implementation of the guidelines is limited by a lack of training in their use (39). There is a general lack of health professionals providing rehabilitation for osteoarthritis, and graduates are ill-prepared, especially in rural settings (40).

In Brazil, there are no formal osteoarthritis CPGs endorsed by the Ministry of Health and the information available to health professionals and people with osteoarthritis is generally low-quality. A survey of Brazilian physiotherapists highlights a serious deficiency in engagement with research evidence to inform practice (28).

A CPG for osteoarthritis has just been released in Nepal (41), hence data on its impact on care is unavailable. Undergraduate and post-graduate medical and allied health programs do not focus on osteoarthritis management. While international osteoarthritis CPGs have moved away from recommending medications and surgeries for first-line management (8), research in Nepal indicates increasing use of such interventions for musculoskeletal conditions (42). This is compounded by the low health literacy of people with osteoarthritis, and biomedical attitudes to osteoarthritis management.

### Opportunities to Improve Osteoarthritis Care

There were three main themes highlighting opportunities to improve osteoarthritis care:

*l*.*Provide high-quality education and training to upskill health professionals*

In South Africa there are existing evidence-based resources, including the “Standard Treatment Guideline and Essential Medicine List” (38), “Practical Approach for Care Kit” (43) and Western Cape “Essential Pain Management” training program. These resources could be promoted on a national level to integrate osteoarthritis care more effectively into existing health services.

In Brazil, public universities focus on primary health care, yet osteoarthritis is neglected as it is considered a less important condition. There is an opportunity for public universities in Brazil to lead the inclusion of evidence-based osteoarthritis care in curricula. Similarly, professional societies should lead education and training for qualified health professionals and could also play an important role in consumer osteoarthritis education. Existing consumer support groups recognized by the Brazilian Society of Rheumatology could be expanded to facilitate education programs for consumers (44).

Recently, Nepali clinician-researchers have received grants from the International Association for the Study of Pain to develop education and training for physiotherapists delivering care for pain conditions, including osteoarthritis (45). The recently published osteoarthritis guideline (41) will go some way to support healthcare professionals to deliver best-evidence osteoarthritis care. Future iterations may incorporate international campaigns such as “Choosing Wisely” that aim to educate both clinicians and public on the “right” care for osteoarthritis (46).

*ll*. *Leverage current national health priorities in non-communicable diseases*

With the advent of aging populations in LMICs, the focus is slowly shifting from communicable diseases to NCDs (27). South Africa has opportunities to adapt successful strategies from other areas of public health to improve osteoarthritis care. For example, improvements were seen in child and maternal health when medical specialists were involved in training local healthcare professionals to transfer skills and improve care (47). A similar model could be used to improve osteoarthritis management.

As major causes of morbidity and mortality in Brazil, cardiovascular disease and diabetes are priority conditions for the healthcare system. There is potential to leverage funding for these conditions on the basis that they share risk factors (e.g., obesity) and lifestyle interventions are key to maintaining health (physical activity/exercise and maintaining healthy weight). Further, the prevalence, severity and cost of osteoarthritis are projected to increase enormously in the coming decade if the current levels of obesity are not urgently addressed and reduced (48).

Encouragingly, the Nepal Health Research Council recently added osteoarthritis as a priority research area, in response to advocacy and calls for prioritizing musculoskeletal pain conditions (30). This is a promising step toward improving both the quality and quantity of osteoarthritis research in Nepal.

*lll*. *Leverage existing resources and innovations*

Existing health innovations and technologies could be expanded to improve osteoarthritis care. A technology-based approach in South Africa that could be expanded for osteoarthritis care is the “Vula application.” This application provides instant referrals from primary care clinicians to specialists with a chat function. This communication channel reduces “wastage” in referral pathways. For example, premature referrals for surgical management could be avoided and instead, a specialist can advise the referring doctor how to optimize care at the community level, in real time.

Mobile health technologies are a means to reach a large population of people with osteoarthritis, especially those outside of urban areas (49). Telehealth has been used in South Africa since the 1970s, but only for certain conditions and regions (50). Although a survey by the Medical Protection Society found that doctors were seeing the benefits of telehealth, 9/10 were concerned that some patients might be left behind (51). Telehealth for knee osteoarthritis has been shown to be feasible in Brazil (52).

An innovative approach is currently being trialed in Nepal to improve osteoarthritis care through improved training for physiotherapists in pain management (45). The project will provide patients with direct access to physiotherapists, who have been trained in delivering best evidence osteoarthritis care. This will bypass surgeons who can then focus their practice on those who need surgical care (e.g., trauma or advanced stage osteoarthritis).

## Discussion

### Considerations for Future Implementation of Best Evidence OA Care

**Figure 2:**
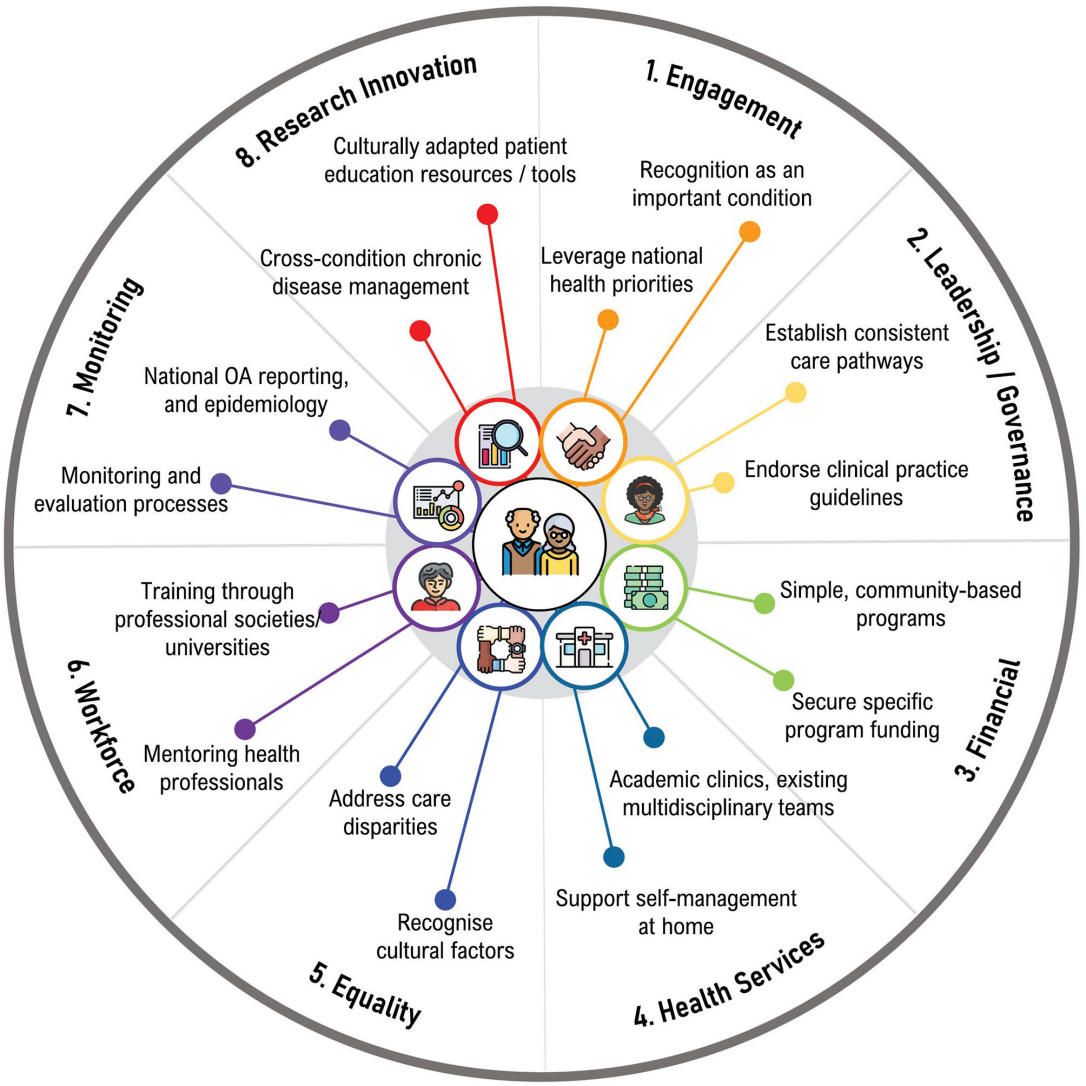
Considerations for implementation of best-evidence osteoarthritis care in Lowand Middle-Income Countries.

Considerations for the implementation of best evidence osteoarthritis care in South Africa, Brazil, and Nepal are discussed below, and categorized according to the GMUSC pillars, which is summarized in Figure 2.

#### South Africa

In South Africa, it is important to advocate with policy makers to ensure osteoarthritis is included on the burden of disease list, particularly given the economic impacts attributable to osteoarthritis (pillar 1). There should be resuscitation of conversation between the South African Rheumatism and Arthritis Association and government about prioritizing osteoarthritis care, as advocated in the “Bone and Joint Decade” (pillar 1). Similarly, pressure should be applied to professional societies and universities to prioritize osteoarthritis and include best evidence osteoarthritis care training in their curricula (pillars 1, 6). A continuum of care pathway should be established for osteoarthritis across all levels of care (pillars 2, 4), with monitoring and evaluation (pillar 7). Implementation strategies should start small, working at the community and family level, and with allied health practitioners (pillars 1, 4, 6). There should also be recognition and inclusion of traditional healers, alternate, and complementary medicine healthcare practitioners in any osteoarthritis education and skills training (pillars 4, 6) (53).

#### Brazil

Broad scale implementation of coordinated osteoarthritis management programs is currently unrealistic in Brazil, considering the heterogeneity in healthcare between Brazilian regions (54) (pillar 5). Strategies to implement best evidence osteoarthritis care should be simple, short duration, low cost, and emphasize self-management at home (pillars 3, 4). Small implementation initiatives that include mentoring for health professionals would be a good place to start (pillar 6). These could be initiated in academic clinics that can provide a multidisciplinary team, but funding should be secured for greater impact (pillars 3, 4). In seeking funding, it is important to connect osteoarthritis management with cardiovascular disease, obesity, and diabetes, given the synergies of risk factors and emphasis on lifestyle interventions (pillars 1, 3). Addressing these conditions together could imply less cost and more benefits for any individual condition and is an important area for future research (pillar 8).

#### Nepal

There have been positive steps taken toward improving osteoarthritis care, including the publication of the CPG for osteoarthritis (41) (pillar 6). Future iterations of the guideline may also explicitly list treatments that are recommended against. Culturally adapted patient educational resources should also be developed to support education for self-management (pillars 1, 8), as it has been recently completed for people with back pain (42). This approach is also likely to improve the quality of, and standardize the advice provided to patients (pillars 1, 4, 6). Similarly, culturally appropriate and adapted patient decision aids and outcome measurement instruments may promote shared decision making (55), and improve tracking of osteoarthritis health outcomes (pillars 1, 4, 7, 8).

There are many challenges to the implementation of best-evidence osteoarthritis care in LMICs, but there are also opportunities for future improvements through advocacy with policy makers, leveraging existing resources, adapting strategies used successfully in other health conditions and providing education for health professionals and people with osteoarthritis. All eight pillars for strengthening health systems can be addressed by the strategies identified in this review, and this framework could be useful in future work to improve osteoarthritis care in LMICs (56).

## Author Contributions

JE, SS, JB, and DH contributed to the conception and design of the study. SS, RT, and MN developed the initial materials they presented at the workshop. JE and JB conducted the thematic analysis. JE and SS wrote the first draft of the manuscript. JB, RT, and MN wrote sections of the manuscript. All authors contributed to the literature review, manuscript revision, read, and approved the submitted version.

## Funding

DH is supported by a National Health and Medical Research Council Leadership 2 Investigator Grant APP1194737.

## Conflict of Interest

The authors declare that the research was conducted in the absence of any commercial or financial relationships that could be construed as a potential conflict of interest.

## Publisher's Note

All claims expressed in this article are solely those of the authors and do not necessarily represent those of their affiliated organizations, or those of the publisher, the editors and the reviewers. Any product that may be evaluated in this article, or claim that may be made by its manufacturer, is not guaranteed or endorsed by the publisher.
